# Effect of an internet- and app-based stress intervention compared to online psychoeducation in university students with depressive symptoms: Results of a randomized controlled trial^☆^

**DOI:** 10.1016/j.invent.2021.100374

**Published:** 2021-02-24

**Authors:** Mathias Harrer, Jennifer Apolinário-Hagen, Lara Fritsche, Christel Salewski, Anna-Carlotta Zarski, Dirk Lehr, Harald Baumeister, Pim Cuijpers, David Daniel Ebert

**Affiliations:** a Clinical Psychology and Psychotherapy, Friedrich-Alexander-University Erlangen-Nuremberg, Erlangen, Germany; b Institute of Occupational, Social and Environmental Medicine, Heinrich-Heine-University Düsseldorf, Düsseldorf, Germany; c Department of Health Psychology, Institute for Psychology, University of Hagen, Hagen, Germany; d Division of Online Health Trainings, Innovation Incubator, Leuphana University, Lüneburg, Germany; e Clinical Psychology and Psychotherapy, Institute of Psychology and Education, Ulm University, Ulm, Germany; f Department of Clinical, Neuro, and Developmental Psychology, Vrije Universiteit Amsterdam, Amsterdam, the Netherlands

**Keywords:** Perceived stress, Randomized controlled trial, College students, Depression, Internet intervention, App

## Abstract

Depression is highly prevalent among university students. Internet-based interventions have been found to be effective in addressing depressive symptoms, but it is open if this also applies to interventions directed at academic stress. It is also largely unclear if the techniques employed in such programs provide significant additional benefits when controlling for non-specific intervention effects.

A sample of *N* = 200 students with elevated levels of depression (CES-D ≥ 16) of a large distance-learning university were randomly assigned to either an Internet- and App-based stress intervention group (IG; *n* = 100) or an active control group (CG; *n* = 100) receiving an Internet-based psychoeducational program of equal length. Self-report data was assessed at baseline, post-treatment (7 weeks) and three-month follow-up. The primary outcome was depression (CES-D) post-treatment. Secondary outcomes included mental health outcomes, modifiable risk factors, and academic outcomes.

We found significant between-group effects on depressive symptom severity (*d* = 0.36; 95% CI: 0.08–0.64), as well as behavioral activation (*d* = 0.61; 95% CI: 0.30–0.91), perceived stress (*d* = 0.45; 95% CI: 0.18–0.73), anxiety (*d* = 0.35; 95% CI: 0.03–0.67) and other secondary outcomes post-treatment. Effects on depression were sustained at three-month follow-up. Response rates for depressive symptoms were significantly higher in the IG (26%) than the CG (14%) at post-test (*χ*^2^=4.5, *p* = 0.04), but not at three-month follow-up (*p* = 0.454). We also found significant effects on relevant academic outcomes, including work impairment (follow-up; *d* = 0.36), work output (post-treatment; *d* = 0.27) and work cutback (follow-up; *d* = 0.36).

The intervention was more effective for depressive symptoms compared to the CG, and so controlling for unspecific intervention effects. This suggests that specific techniques of the intervention may provide significant additional benefits on depressive symptoms.

Trial registration: German Clinical Trial Registration (DRKS): DRKS00011800 (https://www.drks.de/drks_web/navigate.do?navigationId=trial.HTML&TRIAL_ID=DRKS00011800).

## Highlights

• Beneficial effects of Internet stress intervention on depression compared to psychoeducation
• Adherence to and acceptance of the intervention was high
• Most participants were first-time help-seekers
• Results suggest specific effects of intervention techniques beyond general intervention effects
• Internet stress interventions could be alternative way to effectively reduce depressive symptoms

## 1 Introduction

Depressive disorders are very common in university student populations, with twelve-month estimates ranging from 4.5% to 18.5% (Auerbach et al., 2016, Auerbach et al., 2018). Suffering from mental disorders such as depression in the university years is associated with numerous negative outcomes for both the individual and society, including lower college retention and academic performance (Breslau et al., 2008; Eisenberg et al., 2009a; Hysenbegasi et al., 2005; Ishii et al., 2018; Kessler et al., 1995), higher disability (Alonso et al., 2018), as well as worse social functioning (Goldman-Mellor et al., 2014; Niederkrotenthaler et al., 2014) in later life. Addressing depressive symptoms in university students through early intervention is thus of paramount importance.

Within the university student population, distance-learning students may be particularly at risk of developing mental health problems. Distance-learning institutions are more frequently used by older individuals, parents, or by employees attaining additional qualifications besides holding a job (Simpson, 2013; Stoessel et al., 2015), all of which may result in a more stressful learning environment for affected students (Apolinário-Hagen et al., 2018). Results from a large German survey among 5721 students suggest that distance learners face great strain compared to on-site students, likely due to having to meet the demands of multiple societal roles (Apolinário-Hagen et al., 2018). Resulting mental health problems in distance-learning students have been associated with worse academic attainment (Richardson, 2015).

There is a large unmet need for treatment in university students. Estimates of overall 12-month treatment rates among students with depression range from 30.2 to 43.9% (Bruffaerts et al., 2019; Eisenberg et al., 2011). Lack of time, personal stigma, and preference to self-manage have been reported as important treatment barriers in university student populations (Czyz et al., 2013; Eisenberg et al., 2009b; Ennis et al., 2019; Miranda et al., 2015).

Internet-based psychological interventions are increasingly recognized as a promising way to address mental health problems and facilitate help-seeking in tertiary education settings (Davies et al., 2014; Ebert et al., 2017a; Harrer et al., 2018a). Internet-based programs can be accessed easily and anonymously, and provide high scalability (Ebert et al., 2019). Implementation of Internet-based services may be particularly helpful for distance-learning universities, where students commonly do not have direct access to on-site student mental health services. Recent developments associated with the COVID-19 pandemic, seen by some as a “black swan” for mental health care (Wind et al., 2020), have further underlined the relevance of Internet-based mental health services, both for “traditional” and distance-learning university students.

Although Internet-based programs show the potential to overcome some of the treatment barriers of traditional mental health services, successful implementation into routine practices remains challenging (Gilbody et al., 2015; Mohr et al., 2017). While there is evidence that Internet-based interventions directed at university students can be successfully implemented into routine care (Dear et al., 2019), many pragmatic trials implementing Internet-based treatment in naturalistic settings report relatively low uptake rates, varying between 3 and 25% (Kaltenthaler et al., 2008; Lillevoll et al., 2014; Whiteside et al., 2014; Woodford et al., 2011).

This underscores the importance of providing formats which are not only effective, but also catch the interest of the target population. In a survey of distance-learning students, interventions for relaxation and stress management were the most sought after, with 66.9% and 54.8% indicating potential interest in participating (Apolinário-Hagen et al., 2018). Internet-based stress interventions are also frequently used by students with clinically relevant levels of depression who did not previously seek help through conventional health care channels (Harrer et al., 2018b). Provision of Internet-based stress interventions could therefore be a non-stigmatizing approach to increase treatment utilization among students with depressive symptoms.

Internet-based stress management interventions (i.e. interventions which convey techniques to cope with modifiable or non-modifiable stressors) have not only been found to be effective in reducing perceived stress (Heber et al., 2017), but have also shown moderate to high effects on depressive symptoms (Cohen's *d* = 0.52–0.95; Ebert et al., 2016b; Ebert et al., 2016a; Harrer et al., 2018b; Heber et al., 2016), even in participants with clinically relevant symptoms of depression (*d* = 0.67–1.19; Harrer et al., 2018b; Weisel et al., 2018). These effects are comparable to the ones of evidence-based psychotherapies for depression (*d* = 0.62–0.92; Barth et al., 2016).

Internet-based interventions have been shown to be effective in preventing (Buntrock et al., 2016; Ebert et al., 2017a) and treating (Königbauer et al., 2017) depression. Yet, no prospective study has so far examined if Internet-based interventions for academic stress also have this potential in students with elevated depression levels. It is well established that perceived stress contributes to the development of depression (Cohen et al., 2007), and negatively influences its clinical course (Hammen, 2005; Mazure, 1998). Intervention approaches that exploit this strong interconnection between stress and depression, however, remain understudied. If found to be effective, Internet-based stress intervention may be used as an alternative way to address depressive symptoms. In particular, they may be implemented to decrease the treatment gap among students with depression who would not consider conventional depression treatment.

Furthermore, while there is substantial evidence that Internet-based stress interventions can be effective when compared to inactive control groups (Harrer et al., 2018a, Harrer et al., 2018b; Heber et al., 2017), it is less clear if such interventions are also superior to control groups in which participants are actively engaged as well. Thus, it remains largely unknown if stress interventions have an incremental “verum” effect associated with applied strategies beyond known common factors (e.g. positive regard, expectations, learning; Cuijpers et al., 2019).

In this study, we therefore aim to evaluate the effectiveness of an Internet- and App-based stress intervention in distance-learning students with elevated levels of depression. We aim to investigate effects in comparison to an active control group receiving an Internet-based stress psychoeducation program. We hypothesized the Internet-based stress intervention to be more effective when compared to the active control group. Furthermore, to assess the broader impact of the intervention, we also aim to explore effects on various secondary outcomes. This includes symptoms commonly associated with depression (anxiety, perceived stress, worrying, emotional exhaustion, behavioral activation), modifiable risk and protective factors for depression (resilience, emotion regulation, self-compassion, self-esteem, beliefs about stress), as well as, considering the deleterious effect of mental disorders on academic achievement, effects on work impairment and academic productivity.

## 2 Materials and methods

The trial investigated in this study has been registered in the German clinical trials register (DRKS00011800). The study proceedings were approved by the University of Erlangen-Nuremberg ethics committee (Erlangen, Germany; 33_17Bc). Furthermore, a protocol detailing the methods of this trial has been published (Harrer et al., 2019). We present the methods and results of this study in accordance to the CONSORT Statement (Moher et al., 2010), and the Guidelines for Executing and Reporting Research on Internet Interventions (Proudfoot et al., 2011). The code used for the analyses in this study has been made openly available in an Open Science Framework (OSF) repository (www.osf.io/6y9tq/).

### 2.1 Design

We conducted a two-armed randomized controlled trial (RCT) with two conditions. The intervention group (IG; *n* = 100) received *StudiCare Fernstudierende*, an Internet-based stress management intervention. The active control group (CG; *n* = 100) received an Internet-based psychoeducation program. The sample size (*N*_total_ = 200) of the trial allows to detect a between-group effect size of *d* = 0.40 with a power (1 − *β*) of 80%, and a two-sided alpha of 0.05. A recent meta-analytic review for Internet-based stress interventions reported an effect size of *d* = 0.43 for perceived stress, and a somewhat smaller effect of *d* = 0.34 on depressive symptoms (Heber et al., 2017). Results for Internet-based interventions addressing psychological distress in tertiary education are mixed, ranging from non-significant findings to moderate-sized effects in favor of the intervention (Cavanagh et al., 2013; Chiauzzi et al., 2008; Day et al., 2013; Frazier et al., 2015; Hintz et al., 2015). An effect size of *d* = 0.40 was therefore assumed for sample size calculations. Participants were assessed at baseline (T1), post-treatment (T2; seven weeks after randomization), and three-month follow-up (T3). More details on the study design are described in the study protocol (Harrer et al., 2019).

### 2.2 Participants

Participants were included when they (i) showed elevated levels of depression, defined by a score of ≥16 on the 20-item German version of the Center for Epidemiological Studies' Depression Scale (CES-D; Hautzinger et al., 2012; Radloff, 1977). Such scores indicate subthreshold to full symptoms of depression during the last two months. Participants also had to (ii) be enrolled at in a bachelor's, master's, or university diploma (“Diplom”; corresponds to a master's degree) program at a large German distance-learning university (*FernUniversität in Hagen*) at the beginning of the intervention, (iii) be at least 18 years old, (iv) have Internet access, (v) declare willingness to provide self-report data at all three assessment points, and (vi) give informed consent.

Participants were excluded when they (i) reported dissociative symptoms or psychosis (currently or in the past), and/or (ii) showed a considerable suicide risk, defined as a score >1 on item nine of the German version of the Beck Depression Inventory (BDI-II; Hautzinger et al., 2006; “I feel I would be better off dead”, or “I would kill myself if I had the chance”). Individuals defined as showing an elevated risk for suicide were given detailed information about treatment options for depression. They were also asked to see their physician or a psychiatrist as soon as possible to initiate psychiatric or psychotherapeutic treatment. There was no monetary compensation for participating in one of the interventions.

### 2.3 Recruitment

Participants were recruited in German-speaking countries through information letters distributed through the distance-learning university's mailing list, university press reports and social media insertions. Students were referred to a website created for the intervention. This website contained a registration form through which potential participants could declare interest in partaking in the study.

### 2.4 Eligibility assessment and randomization

Individuals who declared interest in participation were sent an online letter with comprehensive information about the study procedures. Individuals were also asked to fill out an online screening questionnaire to determine their eligibility for the study. Study administration staff checked the answers provided in the screening within 1–2 work days. Individuals who fulfilled all inclusion and none of the exclusion criteria were then asked to provide informed consent and fill out the baseline survey.

In the next step, participants were randomly allocated to the IG or CG. For randomization, we used a 1:1 ratio and block size of two in an automated computer-based random integer generator (*Randlist*, Datinf GmbH, Tübingen, Germany). Randomization was conducted by a researcher who was not otherwise involved in the study. During the randomization procedure, allocation was concealed from participants, recruitment staff and e-coaches.

### 2.5 Interventions

#### 2.5.1 StudiCare Fernstudierende

The program evaluated in the IG is an adaptation of *StudiCare Stress* (Harrer et al., 2018b), an Internet- and App-based intervention for college students. Both programs are based on *Get.On Stress*, an Internet-based stress intervention for employees (Ebert et al., 2014; Ebert et al., 2016b; Ebert et al., 2017b; Heber et al., 2013, Heber et al., 2016; Weisel et al., 2018). In its contents, *StudiCare Fernstudierende* only deviates minimally from *StudiCare Stress*, with a few changes made to tailor the intervention more to distance-learning university students' needs. The intervention was delivered using the *Minddistrict* e-Health platform (Minddistrict GmbH, Berlin, Germany). A detailed summary of the content changes and intervention components can be found in the study protocol (Harrer et al., 2019).

The intervention contains seven modules and one booster module (see Table 1). Completion of one module is estimated to take between 30 and 90 min. The intervention contains two main components, the first focusing on problem-oriented coping, and the second focusing on emotion-oriented coping through emotion regulation strategies.

**Table 1:** Modules of the Internet-based Stress Intervention (IG) and the Internet-based psychoeducation program (active CG).

Module	*StudiCare Fernstudierende*	Psychoeducation
1	Introduction Psychoeducation, information about stress and preview of subsequent sessions	Introduction Prevalence and types of stress; Biological response to stress; Effects of stress on emotions, thought, somatic symptoms
2	Problem-solving Stress management strategies, systematic problem-solving using a 6-step problem solving heuristic	Causes of stress Common stressors among students; Lazarus' transactional model of stress
3	Muscle- and breath relaxation Information on basic principles of muscle and breath relaxation, audio exercises for daily usage	Does stress have the same effect on all individuals? Short and long-term consequences of stress; inter-individual differences in stress response
4	Mindfulness Coping with self-criticism, mindfulness exercises	What effect does stress have on the body? Physiological response to stressors; evolutionary background of stress reactions; stress and performance
5	Acceptance and tolerance Dealing with unsolvable problems, psychoeducation on and exercises for acceptance and tolerance of unpleasant emotions	Cognitive appraisal Common dysfunctional thoughts contributing to perceived stress; 5 steps for cognitive reappraisal
6	Self-compassion Self-criticism in precarious situations, defusion of self-worth and performance, exercises for positive self-support, overcoming dysfunctional perfectionistic thought-action patterns	Coping and resources Typical resources and coping mechanisms for stress
7	My master plan Recognizing physiological warning signs, creating a plan for the future	Health Definition of health and sense of coherence
8	Booster session Further information on self-help and psychotherapy, evaluation of training transfer, recap of all sessions, repetition of previous exercises	Booster session Recap of previous material

Participants are instructed to complete one or a maximum of two modules each week. The intervention is therefore intended to last between five to seven weeks. After module two to six, participants can choose to work on optional mini-modules. These mini-modules cover information and exercises on student-specific topics of interest: social support, rumination and worrying, time management, procrastination, test anxiety, sleep, motivation, nutrition and exercise, dealing with writer's block and concentration.

Some features were added to facilitate the transfer or learned strategies into everyday life. A diary App could be downloaded to track mood fluctuations, monitor behaviors influencing one's stress levels, and reflect on intervention strategies that can be implemented into one's daily life routines. The diary contained standardized free-text fields and rating scales. On demand, participants in the IG could also receive automatic messages containing short, motivational prompts via SMS.

#### 2.5.2 Psychoeducation

Participants in the active CG received an Internet-based psychoeducation program. The program primarily covers the cognitive, emotional and physical determinants, symptoms, outcomes of and strategies against psychosocial stress in general, and with respect to distance-learning students (see Table 1). The program is delivered on the same platform as *StudiCare Fernstudierende*. It also contains seven modules and a booster session, and is also intended to be completed within five to seven weeks. In contrast to the actual stress intervention, however, the psychoeducation modules were largely text-based. The program was designed to mainly convey helpful information about stress and coping, but did not directly assist in the implementation of strategies for behavior change into daily life.

### 2.6 Guidance

To facilitate adherence to the intervention while minimizing human capital costs, an adherence-focused guidance concept with personalized feedback on demand was employed. A detailed description of this guidance approach and its theoretical underpinnings can be found in previous literature (Ebert et al., 2014; Ebert et al., 2016a; Zarski et al., 2016).

Guidance in the IG consisted of three parts: (i) monitoring adherence to the intervention, (ii) sending standardized motivational messages after every module, and (iii) providing feedback on demand. Adherence monitoring included sending reminders to participants who had not completed a module within seven days. The standardized motivational messages were sent when a participant completed one of the main modules. They summarized the content of the module and motivated trainees to remain engaged. Feedback on demand was provided within a maximum of 48 h through the internal messaging system of the intervention platform when requested.

Guidance for participants in the active CG included parts (i) and (ii), but did not include feedback on demand.

### 2.7 Primary outcome

The primary outcome were symptoms of depression at T2, measured by the German version of the Center for Epidemiological Studies' Depression Scale (CES-D) 20-item version (ADS; Hautzinger et al., 2012; 20 items; range 0–60; retrospective timeframe of two weeks). Higher CES-D scores indicate greater depression severity. The scale has a high retest reliability of *r*_*tt*_ = 0.81, pointing at the high internal validity of this instrument (Hautzinger et al., 2012). The scale has an excellent level of internal consistency in this study, as indicated by a Cronbach's alpha of 0.91.

### 2.8 Secondary outcomes

Unless otherwise specified, all outcomes were measured for a retrospective timeframe of two weeks.

#### 2.8.1 Mental Health

Secondary mental health outcomes included behavioral activation, rumination and functional impairment as measured by the Behavioral Activation for Depression Scale (BADS; Fuhr et al., 2016; 25 items; range 0–150), perceived stress as measured by the Perceived Stress Scale 10-item version (PSS-10; Cohen et al., 1983; 10 items, range 0–40), state anxiety (short form of the Spielberger State-Trait Anxiety Inventory, STAI-6; Laux et al., 1981; Marteau and Bekker, 1992; 6 items, range 6–24; *at the moment*), worrying (Academic Worrying Questionnaire; AWQ; Wolitzky-Taylor and Telch, 2010; 10 items; range 0–40) and emotional exhaustion (emotional exhaustion subscale of the Maslach Burnout Inventory student version; MBI-S; Gumz et al., 2013; 5 items, range 5–30).

#### 2.8.2 Academic outcomes

To assess effects on academic productivity, we administered the Presenteeism Scale for Students' (PSS; Matsushita et al., 2011) subscale for work impairment (Work Impairment Scale; WIS; 10 items; range 10–50). Productivity losses were assessed by an adaption of the PSS' work output scale (WOS), in which participants indicated the degree to which they were able to reach their usual academic productivity. The rating was given on a visual analog scale ranging from 0% = *completely unproductive* to 100% = *full productivity*. As part of the PS-S, we also assessed the time which students lost at university due to their mental health problems within the last two weeks (in hours). Lastly, college self-efficacy was assessed by the College Self-Efficacy Inventory (CSEI; Solberg et al., 1993; 13 item; range 13–65).

#### 2.8.3 Modifiable risk and protective factors

We also included assessments of several modifiable risk and protective factors for mental illness. We assessed resilience as measured by the Connor-Davidson Resilience Scale short form (CD-RISC; Connor and Davidson, 2003; 2 items; range 0–8), emotion regulation competencies (German version of the Assessment of Emotion Regulation Skills; SEK-27; State Version; Berking and Znoj, 2008; 27 items; range 27–108), self-compassion (Self-Compassion Scale; SCS-D; Hupfeld and Ruffieux, 2011; 12 items; range 12–60), and self-esteem as measured by the Rosenberg Self-Esteem Scale (RSES; Ferring and Filipp, 1996; 10 items; range 10–40). Personal beliefs about the controllability and the harmful and positive nature of stress were assessed using the Beliefs about Stress Scales' (BASS; Laferton et al., 2016) subscales for positive (4 items; range 4–16), negative (8 items; range 8–32) and controllability beliefs (3 items; range 3–12).

#### 2.8.4 Additional measures

Additional questionnaires included demographic variables (assessed at T1 only) and client satisfaction with the intervention (Client Satisfaction Questionnaire, adapted to the online context; CSQ-8; Boß et al., 2016; Nguyen et al., 1983; 8 items), which was only assessed at T2.

### 2.9 Statistical analyses

#### 2.9.1 Main effectiveness evaluation

To evaluate the effectiveness of the intervention compared to the active CG, analyses based on the intention-to-treat (ITT) principle were conducted. Analyses were conducted with *R* version 3.5.2 (R Core Team, 2013). Missing data were imputed through multiple Multivariate Imputation by Chained Equations (MICE) with 100 iterations, using the *mice* package (van Buuren and Groothuis-Oudshoorn, 2011).

We tested if the intervention was superior the active control group in terms of effects on participants' depressive symptom severity and secondary outcomes from T1 to T2, and from T1 to T3. We also compared the proportion of participants with reliable response, reliable symptom deterioration, and at least 50% symptom reduction between the IG and active CG at T2 and T3. A significance level of 0.05 (two-sided) was used for all analyses.

Differences in effects between the two study conditions were assessed using univariate analysis of covariance (ANCOVA). T1 scores of each outcome were used as the covariate. We used two ANCOVA models instead of repeated measures ANOVA in order to test between-group differences separately at T2 and T3. Models were fitted in each of the multiply imputed datasets, and model estimates were then aggregated with Rubin's rules (Barnard and Rubin, 1999) using the *miceadds* and *mitml* package (Grund et al., 2019). To calculate the between-group standardized mean difference (viz. Cohen's *d*) as an effect size, we fitted a linear model with a group term only in the multiply imputed datasets, pooled the unstandardized group coefficient using Rubin's rules, and then standardized it using the pooled outcome standard deviation. For the primary outcome, we also calculated the within-group effect sizes for both groups. We used the formula by Becker (1988), which controls for the fact that within-group Cohen's *d* are based on correlated data. Following Cohen (1988), *d* = 0.2 can be considered a small effect, *d* = 0.5 a medium and *d* = 0.8 a large effect.

To determine if the depressive symptoms of a participant (as measured by the CES-D) had reliably decreased, we coded the participants as responders or non-responders using the Reliable Change Index (RCI; Jacobson and Truax, 1991). We compared the proportions of reliable responders in the IG and the active CG at T2 and T3 using *χ*^2^-Tests. We also calculated the Number Needed to Treat (*NNT*) to achieve one additional person responding to the intervention compared to psychoeducation. Using the RCI, we determined cases with a reliable depressive symptom deterioration at T2 and T3, and evaluated differences between the IG and active CG using *χ*^2^-Tests. Lastly, we determined the number of participants in both groups who achieved a reduction of >50% in depressive symptoms from T1 to T2 and T3, respectively. Group differences were also compared using *χ*^2^-Tests.

#### 2.9.2 Sensitivity analyses

We conducted two sensitivity analyses to evaluate the robustness of our results. First, we conducted a study completer analysis based on the sample of participants who provided data at all three assessment points. Analyses followed the same procedure as the main effectiveness analysis. In this study, a relatively large number of secondary outcomes were included, thus increasing the risk of alpha error inflation due to multiple testing (Tyler et al., 2011). An appropriate way to avoid potential alpha error inflation is to use joint modeling approaches (Teixeira-Pinto et al., 2009). As a sensitivity analysis, we therefore also fitted a multivariate Bayesian regression model using the *brms* package (Bürkner, 2017) in which all outcomes at T2 and T3, respectively, were estimated jointly, controlling for the T1 scores of each included outcome. This approach has several advantages, including that the correlation between outcomes is explicitly modeled, and that a straightforward estimation of aggregated parameter estimates in multiply imputed datasets is possible by combining the posterior distributions (Bürkner, 2019).

#### 2.9.3 Subgroup analysis

To estimate the effects of the intervention in participants with full-symptom depression, we conducted a subgroup analysis in which only students with a score of CES-D ≥ 20 at T1 were included. A score of 20 on the CES-D has been shown to be valid cut-off to detect major depression in the general population (Vilagut et al., 2016). The same analyses as in the main effectiveness analysis were conducted.

#### 2.9.4 Process evaluation

To assess user satisfaction with the stress intervention delivered in the IG and the psychoeducation program in the CG, CSQ-8 data obtained at T2 was examined item-wise. Acceptance of stress intervention modules was analyzed using the module feedback of IG participants. Adherence was assessed for both the IG and active CG by analyzing intervention completion rates tracked within the intervention platform. Lastly, we also analyzed the proportion of participants who accessed the diary App in the IG.

## 3 Results

**Fig. 1:**
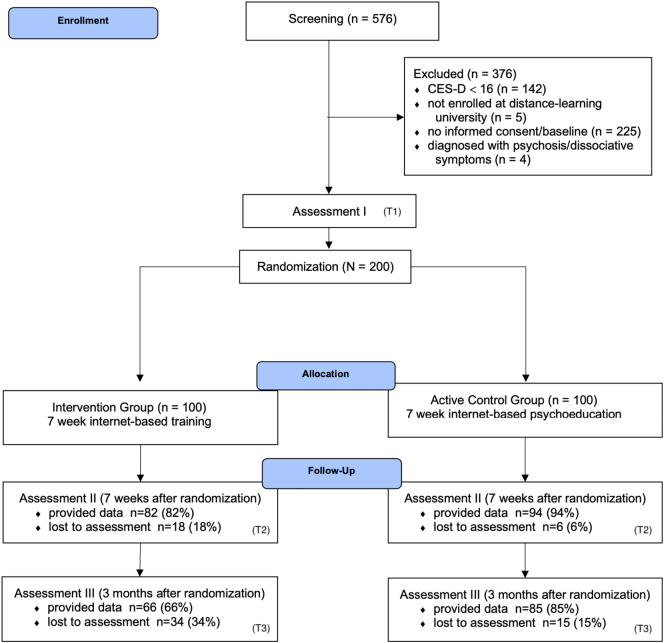
Study flow.

Recruitment for the study began in April 2017. The last follow-ups were completed on May 6, 2019. The study flow is depicted in Fig. 1. In the active CG, we could not obtain follow-up data from six participants (6%) at T2, and 15 (15%) at T3. In the IG, 18 (18%) and 34 (34%) participants were lost to follow-up at T2 and T3, respectively. Demographic data of the included participants are summarized in Table 2. The sample had a mean age of 36.97 (*SD*: 9.52). This is higher than the “usual” college age, but representative of the student body at the distance-learning university at which recruitment took place. About two thirds (*n* = 129; 64.5%) of the participants reported that they had not previously consulted a physician, psychotherapist or counselor for their mental health problems, and can thus be considered first-time help-seekers. Descriptive data for all outcomes at all three assessment points is shown in Table 3.

**Table 2:** Baseline characteristics.

Characteristics	All participants (*N* = 200)	Intervention (*N* = 100)	Active control (*N* = 100)
*Sociodemographics*			
- Age, *M* (*SD*)	36.97 (9.52)	37.53 (9.53)	36.40 (9.52)
- Gender, female, *n* (%)	170 (85)	85 (85)	85 (85)
- In a relationship, *n* (%)	148 (74)	68 (68)	80 (80)
- Married, *n* (%)	89 (44.5)	40 (40)	49 (49)
- Children, yes, *n* (%)	83 (41.5)	45 (45)	38 (38)
- Employed, *n* (%)	163 (81.5)	82 (82)	81 (81)

*Studies*			
- Computer Science, *n* (%)	17 (8.5)	7 (7)	10 (10)
- Economics, *n* (%)	27 (13.5)	10 (10)	17 (17)
- Education, *n* (%)	19 (9.5)	9 (9)	10 (10)
- Humanities, *n* (%)	6 (3)	3 (3)	3 (3)
- Law, *n* (%)	16 (8)	13 (13)	3 (3)
- Mathematics, *n* (%)	2 (1)	0 (0)	2 (2)
- Social Science, *n* (%)	113 (56.5)	58 (58)	54 (54)
- Bachelor's Program, *n* (%)	149 (74.5)	78 (78)	71 (71)
- Master's/Diploma Program, *n* (%)	51 (25.5)	22 (22)	29 (29)
- Semester (current program), *M* (*SD*)	5.49 (4.00)	5.69 (3.99)	5.29 (4.02)
- Semester (total), *M* (*SD*)	12.22 (7.58)	12.67 (7.43)	11.77 (7.75)

*Living*			
- Alone, *n* (%)	69 (34.5)	35 (35)	34 (34)
- With partner/parents/flat share, *n* (%)	131 (65.5)	65 (65)	66 (66)

*Main source of funding*			
- Job, *n* (%)	144 (72)	70 (70)	74 (74)
- Loan, *n* (%)	3 (1.5)	3 (3)	0 (0)
- Parents/partner/relatives, *n* (%)	39 (19.5)	16 (16)	23 (23)
- Other, *n* (%)	14 (7)	11 (11)	3 (3)

*First-time help seeker*			
- Yes, *n* (%)	129 (64.5)	63 (63)	66 (66)

**Table 3:** Descriptive statistics, between-group effect sizes and results of analyses of covariance (ANCOVA) based on the intention-to-treat sample for the primary and secondary outcomes.

Outcome and assessment point	Active control (*n* = 100)		Intervention (*n* = 100)		Effect size		ANCOVA	
	*M*	*SD*	*M*	*SD*	*d*	95% CI	*F*	*p*
*Primary outcome*								
Depression (CES-D, 0–60)								
- T1 (baseline)	25.05	7.85	23.60	8.17				
- T2 (7 weeks)	21.91	10.30	18.18	10.09	0.36	0.08–0.64	7.83	0.005
- T3 (3 months)	18.96	11.30	15.53	10.23	0.31	0.04–0.59	5.64	0.018

*Mental health*								
Behavioral Activation for Depression (BADS, 0–150)								
- T1 (baseline)	82.93	22.92	82.34	20.34				
- T2 (7 weeks)	89.10	23.93	103.66	21.93	0.61	0.30–0.91	17.12	<0.001
- T3 (3 months)	91.11	31.99	105.37	26.37	0.47	0.14–0.81	7.33	0.019
Perceived stress (PSS-10, 0–40)								
- T1 (baseline)	24.08	5.37	23.57	5.92				
- T2 (7 weeks)	20.83	6.99	17.53	7.28	0.45	0.18–0.73	13.10	<0.001
- T3 (3 months)	19.75	9.37	17.20	8.03	0.30	−0.02–0.59	3.79	0.057
Anxiety (STAI-6, 6–24)								
- T1 (baseline)	14.98	3.75	14.59	3.37				
- T2 (7 weeks)	14.23	4.04	12.80	3.89	0.35	0.03–0.67	4.88	0.039
- T3 (3 months)	13.05	4.34	12.39	4.56	0.15	−0.19–0.49	0.26	0.614
Worrying (AWQ, 0–40)								
- T1 (baseline)	17.55	5.16	16.74	4.85				
- T2 (7 weeks)	17.29	5.86	15.18	6.14	0.35	0.06–0.63	7.65	0.007
- T3 (3 months)	15.37	7.50	13.67	6.38	0.25	−0.04–0.53	3.23	0.073
Emotional exhaustion (MBI-S, 5–30)								
- T1 (baseline)	19.50	5.66	19.39	5.10				
- T2 (7 weeks)	18.19	6.06	17.74	5.90	0.08	−0.21–0.36	0.22	0.638
- T3 (3 months)	18.34	7.28	14.58	6.59	0.52	0.24–0.80	16.44	<0.001

*Academic outcomes*								
Work impairment (WIS, 10–50)								
- T1 (baseline)	32.33	6.66	32.30	5.65				
- T2 (7 weeks)	31.51	7.77	29.83	7.58	0.22	−0.06–0.49	3.70	0.055
- T3 (3 months)	30.92	7.56	28.09	7.94	0.36	0.05–0.67	6.04	0.017
Work output (WOS, 0–100)								
- T1 (baseline)	54.63	28.08	56.39	25.70				
- T2 (7 weeks)	54.42	29.28	62.34	29.22	0.27	0.00–0.54	4.24	0.039
- T3 (3 months)	56.82	33.71	65.59	31.82	0.27	−0.11–0.64	1.18	0.313
Work cutback (PS-S, h)								
- T1 (baseline)	9.91	14.96	8.02	12.33				
- T2 (7 weeks)	8.33	11.90	5.81	10.16	0.23	−0.06–0.51	2.47	0.117
- T3 (3 months)	11.50	17.82	5.92	12.06	0.36	0.05–0.68	4.80	0.036
College self-efficacy (CSEI, 13–65)								
- T1 (baseline)	46.72	11.48	47.50	11.66				
- T2 (7 weeks)	51.09	13.17	50.11	12.42	−0.08	−0.36–0.21	0.19	0.660
- T3 (3 months)	51.31	18.10	51.17	14.89	−0.01	−0.32–0.31	−0.04	1.000

*Risk and protective factors*								
Resilience (CD-RISC, 0–8)								
- T1 (baseline)	5.64	1.71	5.51	1.60				
- T2 (7 weeks)	5.53	1.82	5.92	1.73	0.22	−0.07–0.51	2.21	0.146
- T3 (3 months)	5.15	2.24	5.93	1.88	0.37	0.08–0.66	7.36	0.007
Emotion regulation competencies (SEK-27, 27–108)								
- T1 (baseline)	73.83	12.70	74.57	13.72				
- T2 (7 weeks)	76.44	14.59	85.35	14.15	0.59	0.32–0.87	24.00	<0.001
- T3 (3 months)	78.83	26.62	82.98	19.22	0.18	−0.13–0.49	1.10	0.298
Self-compassion (SCS-D, 12–60)								
- T1 (baseline)	19.17	7.86	19.68	7.42				
- T2 (7 weeks)	21.37	9.22	26.52	9.36	0.54	0.26–0.81	22.97	<0.001
- T3 (3 months)	20.63	10.99	26.44	9.52	0.54	0.26–0.83	18.93	<0.001
Self-esteem (RSES, 10–40)								
- T1 (baseline)	27.49	6.74	28.79	6.39				
- T2 (7 weeks)	29.20	6.76	31.80	6.24	0.39	0.12–0.67	20.82	<0.001
- T3 (3 months)	29.83	10.13	31.16	8.10	0.14	−0.19–0.48	0.15	0.708
Negative beliefs about stress (BASS, 8–32)								
- T1 (baseline)	26.45	4.16	26.36	3.56				
- T2 (7 weeks)	25.18	4.29	24.73	4.18	0.11	−0.18–0.39	0.70	0.404
- T3 (3 months)	25.63	4.38	23.83	4.00	0.42	0.11–0.73	8.66	0.006
Positive beliefs about stress (BASS, 4–16)								
- T1 (baseline)	8.95	3.25	8.99	2.96				
- T2 (7 weeks)	9.61	3.02	10.45	2.85	0.28	0.01–0.56	5.34	0.022
- T3 (3 months)	9.31	3.13	10.08	3.13	0.24	−0.04–0.53	3.27	0.074
Controllability beliefs about stress (BASS, 3–12)								
- T1 (baseline)	8.08	2.10	8.13	2.05				
- T2 (7 weeks)	8.73	2.04	9.88	1.67	0.59	0.31–0.86	20.83	<0.001
- T3 (3 months)	8.58	2.24	10.12	1.93	0.69	0.40–0.98	19.94	<0.001

### 3.1 Main effectiveness analysis

#### 3.1.1 Depressive symptoms

Results of the ANCOVAs indicated a significant between-group effect on depressive symptoms at T2 (*F* = 7.83, *p* = 0.005) and T3 (*F* = 5.64, *p* = 0.018) favoring the IG. A small between-group effect size was found both at T2 (*d* = 0.36, 95% CI: 0.08–0.64) and T3 (*d* = 0.31, 95% CI: 0.04–0.59). In the IG, we found within-group effect sizes of *d* = 0.61 (95% CI: 0.39–0.83; T2) and *d* = 1.0 (95% CI: 0.73–1.26; T3). Within-group effects in the CG were *d* = 0.36 (95% CI: 0.15–0.57; T2) and *d* = 0.72 (95% CI: 0.47–0.96; T3). Results of the *χ*^2^-Tests revealed that significantly more participants in the IG (*n* = 26) were classified as reliable responders than in the active CG (*n* = 14) at T2 (*χ*^2^=4.5, *p* = 0.042). These results equal a *NNT* of 9 (95% CI: 4.4–96.3). Although more participants in the IG also showed a reliable response at T3 (*n* = 33; 33%) than in the active CG (*n* = 27; 27%), this difference was not significant (*χ*^2^=0.86, *p* = 0.454). Only a very small proportion of participants experienced reliable symptom deterioration at both T2 (IG: *n* = 4, 4%; active CG: *n* = 5, 5%) and T3 (*n* = 3 in both groups; 3%). There were no differences in deterioration rates between both groups at T2 and T3 (both *p* > 0.999). At T2, *n* = 23 participants achieved a 50% reduction in depressive symptoms compared to T1 in the IG, but only *n* = 12 in the active CG. This difference was marginally non-significant (*χ*^2^=4.19, *p* = 0.052). These numbers rose to *n* = 40 (IG) and *n* = 28 (CG) at T3, but the between-group difference did not reach statistical significance (*χ*^2^=3.67, *p* = 0.089).

#### 3.1.2 Secondary outcomes

Results of the secondary outcome analyses are shown in Table 3. ANCOVAs showed significant (*p* < 0.05) effects favoring the IG for the majority of secondary outcomes. Effects ranged from *d* = 0.27 (95% CI: 0.00–0.54) for work output (T2) to *d* = 0.69 (95% CI: 0.40–0.98) for controllability beliefs about stress (T3). Significant between-group effects on controllability beliefs about stress and self-compassion, favoring the IG, were found at both T2 and T3. For perceived stress, anxiety, worrying, work output, emotion regulation competencies, self-esteem and positive beliefs about stress, a significant between-group effect was found at T2, but not at T3. Conversely, for emotional exhaustion, work impairment, work cutback, resilience, and negative beliefs about stress, no significant between-group effect was found at T2, while a significant effect could be detected at T3. For college self-efficacy, no effect was found at both assessment points (T2: *p* = 0.660; T3: *p* > 0.999).

### 3.2 Sensitivity analyses

Detailed results of the study completer analysis are provided in Table S1 in the Supplement. Results of this analysis were similar to the main analysis results, but slightly higher effect sizes were found on the primary outcome at T2 (*d* = 0.43, 95% CI: 0.11–0.75) and T3 (*d* = 0.46; 95% CI: 0.14–0.78). Results of the joint Bayesian model closely mirrored the ones of the main effectiveness analysis (see Table S2, Table S3 and Fig. S1 in the Supplement). However, while the effect on work output at T2 was significant in the main analysis, the 95% credible interval of this estimate in the Bayesian model included zero (*b* = 7.30, 95%CrI: −0.34–14.95).

### 3.3 Subgroup analysis

At T1, 69% (*n* = 138) of the participants showed a score of CES-D ≥ 20, indicating the likely presence of full-symptom major depression (IG: *n* = 66, 66%; active CG: *n* = 72, 72%). Results of analyses conducted in this subgroup were largely comparable to the ones of the main outcome analysis (see Table S4 in the Supplement for detailed results). In this subgroup, we also found small between-group effect sizes at T2 (*d* = 0.37, 95% CI: 0.02–0.72; *F* = 4.52, *p* = 0.035) and T3 (*d* = 0.34, 95% CI: −0.03-0.70). However, we could not ascertain statistical significance for the effect at T3 (*F* = 3.22, *p* = 0.074).

### 3.4 Process evaluation

#### 3.4.1 Adherence to the interventions

On average, participants in the IG completed 5.23 of the seven modules in *StudiCare Fernstudierende*. In the active CG, the mean number of completed psychoeducation modules was 6.51. This equals 74.7% and 93% of the intervention, respectively. Participants in the IG completed optional mini-modules in the majority (65.6%) of sessions in which they were available. A total of 64 participants (64%) in the IG downloaded and logged into the diary App at least once.

#### 3.4.2 Usefulness, difficulty and duration of intervention modules

On average, participants in the IG described the intervention modules as useful and not too difficult (see Table S6 in the Supplement).

#### 3.4.3 Client satisfaction

Participants' satisfaction with the stress intervention was very high. Overall, 95.1% of participants in the IG (*n* = 78) rated the quality of the intervention as good or excellent, and 96.3% (*n* = 79) indicated that the intervention helped them (a great deal) to cope more effectively with their problems. Interestingly, many participants in the active CG were also satisfied with the psychoeducation material they received as an intervention of its own right. In sum, 74.2% (*n* = 69) of the participants in the CG rated the psychoeducation program as good or excellent, and about half (48.4%, *n* = 45) would recommend the program to a friend with similar issues (see Table S6 in the Supplement).

## 4 Discussion

This trial investigated the effectiveness of an Internet-based stress intervention in distance-learning students with elevated levels of depression. We found a greater reduction in depressive symptoms in the intervention group compared to a control group receiving an Internet-based psychoeducation program seven weeks after randomization (T2). These effects were largely sustained at three-month follow-up (T3).

Significant effects were also found for a range of secondary outcomes at T2 and T3. This included benefits on symptoms related to depression, such as anxiety (T2), worrying (T2), emotional exhaustion (T3), perceived stress (T2) and behavioral activation, as well as effects on modifiable risk and protective factors, such as self-compassion, controllability beliefs about stress, or resilience (T3). We also found positive effects on academic outcomes. This indicates that the intervention may be useful to remediate the detrimental effect of depressive symptoms on academic performance. The effect sizes (*d* = 0.27–0.36) are comparable to the one reported by a recent meta-analysis of e-mental health interventions in university students (*g* = 0.26; Bolinski et al., 2020), although this review only included objective measures of academic performance (i.e. exam results, GPA).

At baseline (T1), more than two thirds (69%) of our recruited sample reported depressive symptoms high enough to indicate the likely presence of a full-symptom major depression. Effects in this subgroup were comparable to the ones in the main analysis.

Overall, the intervention was well accepted. With a mean completion rate of 75%, adherence to the intervention was high, and comparable to a prior examination in university students (72%; Harrer et al., 2018a, Harrer et al., 2018b). Like in this previous trial, we also found that the large majority (65%) of distance-learning students included in present study were first-time help-seekers. This underscores the potential of stress interventions to facilitate help-seeking among students with an unmet need for treatment.

The between-group effect on depression in this study at T2 (*d* = 0.36) is smaller than the ones found in previous trials examining similar versions of the intervention in university student and occupational samples with elevated stress (*d* = 0.52–0.95; Ebert et al., 2016b; Ebert et al., 2016a; Harrer et al., 2018a, Harrer et al., 2018b; Heber et al., 2016). It should be noted, however, that these trials used inactive waitlist control groups as the comparator, which can lead to an overestimation of intervention effects (Mohr et al., 2014). In this trial, we tested the intervention's effects against an active control group, which also received extensive seven-week psychoeducation on stress and coping strategies, as well as a rudimentary guidance format. Psychoeducation formats can be an effective intervention for depressive symptoms, with meta-analytic effect size estimates ranging between *d* = 0.20 and 0.65 (Cuijpers, 1998; Donker et al., 2009). In line with this, the psychoeducation format used in this study was well received as an intervention of its own right. Three quarters of CG participants rated its quality as good or excellent, and adherence was high. In summary, this suggests that while Internet-based provision of helpful reading material and standardized feedback alone may already have some positive effect on depressive symptoms, specific techniques of the Internet-based stress intervention provide significant additional benefits. However, it should be noted that we cannot completely rule out that effects in the active CG are based on natural processes, such as spontaneous remission or regression to the mean (Cuijpers et al., 2017).

The between-group effect on depression found in this study is comparable to the results of a previous trial with similar inclusion criteria, evaluating an Internet-based intervention specifically directed at depressive symptoms compared to psychoeducation (*d* = 0.36; Reins et al., 2019). Effects are also comparable to the ones of face-to-face psychotherapies for depression when only high-quality studies without waitlist controls are considered (*g* = 0.38; Cuijpers et al., 2018). Furthermore, the within-group effects we found in the IG (*d* = 0.61–1.0) reach the one of a large-scale phase IV trial which examined an Internet-based anxiety and depression intervention for university students under routine care conditions (*d* = 0.81; Dear et al., 2019). Together, this indicates that the Internet-based stress intervention is a viable alternative to formats directly targeting depressive symptoms.

Several limitations of this trial should be considered. First, study dropout in the IG was relatively high at T3, and somewhat higher than in the CG. While this is a common finding in Internet intervention trials in university student populations (IG completion rates: 43–53%; CG: 58–87%; Cavanagh et al., 2013; Frazier et al., 2015; Harrer et al., 2018b), results at this assessment point should be interpreted with some caution. Second, with 64%, usage of the App-based component in the IG was relatively low, and no adjunct mobile App was provided in the psychoeducation group. Third, while reliable symptom deterioration was found to be rare, we did not assess other potential negative effects associated with the intervention. Fourth, usefulness, difficulty and duration of each module was only assessed and analyzed in the IG. Fifth, participants were included based on a self-report questionnaire cut-off indicating elevated symptoms of depression. We did not assess how many participants fulfilled the diagnostic criteria of a depressive disorder based on clinical interviews. Future studies may investigate if Internet-based stress interventions are also effective in individuals with a diagnosed depressive disorder. Sixth, while we examined academic outcomes in the form of self-assessments, it was not feasible in this trial to include observer-based performance ratings, such as exam results or GPA. Seventh, while results of our trial indicate that the Internet-based stress intervention effectively reduced depression, we did not include a head-to-head comparison with an intervention directly addressing depressive symptoms. Future studies may therefore examine the potential non-inferiority of Internet-based stress interventions compared to depression-specific interventions directly. Lastly, while results of this study provide evidence that specific components used in the intervention may provide additional effects on depressive symptoms, the concrete working mechanisms through which this is achieved remain largely unknown. Additional research is needed to establish which Internet-based stress management techniques have a beneficial impact on depressive symptoms, and why.

## 5 Conclusions

Internet-based stress interventions may be an acceptable and effective way to address depressive symptoms in adult distance-learning students with elevated depression levels. We found a significantly greater symptom reduction in the intervention compared to an active psychoeducation group, indicating that specific components of the stress intervention may contribute additional benefits. The large majority of participants were first-time help-seekers, hinting at the potential of this intervention format to reach out to burdened students with an unmet need for treatment.

## CRediT authorship contribution statement

CS, JAH and DDE obtained funding for this study. DDE and MH contributed to the development of the Internet-based stress intervention. DDE, MH and JAH contributed to the study design. MH, LF and JAH were responsible for the study management. MH conducted the data analyses and drafted the first version of the manuscript. DDE, JAH, LF, DL, AC, HB and PC contributed to further writing of the manuscript. All authors read and approved the final manuscript.

## Declaration of competing interest

DDE reports to have received consultancy fees or served in the scientific advisory board from several companies such as Novartis, Sanofi, Lantern, Schön Kliniken, Minddistrict, and German health insurance companies (BARMER, Techniker Krankenkasse). DDE is a stakeholder of the Institute for health trainings online (GET.ON), which aims to implement scientific findings related to digital health interventions into routine care. HB reports to have received consultancy fees and fees for lectures or workshops from chambers of psychotherapists and training institutes for psychotherapists.

## Appendix A Supplementary data

Supplementary material Image 1
