# Supplementary material for: Effect of an internet- and app-based stress intervention compared to online psychoeducation in university students with depressive symptoms: Results of a randomized controlled trial
Source: Internet Interv. 2021 Feb 24;24:100374. doi: 10.1016/j.invent.2021.100374 (PMC7932886; doi:10.1016/j.invent.2021.100374)

SUPPLEMENT

Effects of an Internet- and App-based Stress Intervention in University Students with Depressive Symptoms: Results of a Randomized Controlled Trial

Table S1. *Between-group Effect Sizes and Results of Analyses of Covariance (ANCOVA) in the Study Completer Sample for the Primary and Secondary Outcomes at Post-Test and Follow-Up.*

Outcome and Assessment Point	Effect Size		ANCOVA	
	d	95%CI	$F_{1,141}$	p
**Primary Outcome**				
Depression (CES-D)				
- Post	0.43	0.11-0.75	9.37	<0.001
- Follow-up	0.46	0.14-0.78	9.23	<0.001
**Mental Health**				
Behavioral Activation for Depression (BADS)				
- Post	0.75	0.44-1.06	29.15	<0.001
- Follow-up	0.63	0.32-0.94	18.80	<0.001
Perceived Stress (PSS-10)				
- Post	0.55	0.23-0.86	15.26	<0.001
- Follow-up	0.59	0.28-0.91	16.91	<0.001
Anxiety (STAI-6)				
- Post	0.44	0.12-0.76	8.83	0.003
- Follow-up	0.40	0.08-0.73	6.29	0.013
Worrying (AWQ)				
- Post	0.47	0.15-0.79	12.91	<0.001
- Follow-up	0.51	0.19-0.83	13.79	<0.001
Emotional Exhaustion (MBI-S)				
- Post	0.15	0.17-0.48	0.89	0.346
- Follow-up	0.77	0.47-1.08	33.65	<0.001
**Academic Outcomes**				
Work Impairment (WIS)				
- Post	0.36	0.04-0.69	7.49	0.007
- Follow-up	0.45	0.13-0.77	9.72	0.002
Work Output (WOS)				
- Post	0.27	-0.06-0.59	3.32	0.070
- Follow-up	0.32	-0.01-0.64	4.67	0.032
Work Cutback (PS-S)				
- Post	0.24	-0.09-0.57	2.22	0.139
- Follow-up	0.47	0.15-0.79	8.62	0.004
College Self-Efficacy (CSEI)				
- Post	-0.06	-0.39-0.27	0.15	0.699
- Follow-up	-0.13	-0.45-0.20	0.57	0.453

**Risk and Protective Factors**				
Resilience (CD-RISC)				
- Post	0.26	-0.07-0.59	3.77	0.054
- Follow-up	0.35	0.03-0.68	6.38	0.013
Emotion Regulation Competencies (SEK-27)				
- Post	0.63	0.32-0.95	27.27	<0.001
- Follow-up	0.05	-0.28-0.37	0.07	0.789
Self-Compassion (SCS-D)				
- Post	0.66	0.35-0.98	35.32	<0.001
- Follow-up	0.53	0.21-0.85	21.11	<0.001
Self-Esteem (RSES)				
- Post	0.47	0.15-0.79	22.78	<0.001
- Follow-up	-0.02	-0.35-0.31	0.01	0.909
Negative Beliefs about Stress (BASS)				
- Post	0.18	-0.15-0.50	1.81	0.181
- Follow-up	0.44	0.12-0.76	8.76	0.004
Positive Beliefs about Stress (BASS)				
- Post	0.31	0.01-0.64	5.04	0.026
- Follow-up	0.31	0.01-0.64	5.35	0.022
Controllability Beliefs about Stress (BASS)				
- Post	0.55	0.23-0.86	12.77	0.000
- Follow-up	0.72	0.41-1.03	23.45	0.000

Table S2. *Results of the joint Bayesian model for the Primary and Secondary Outcomes at Post-Test and Follow-Up.*

Outcome	$\hat{b}$	*SE*	95%CrI
**Primary Outcome**			
Depression (CES-D)			
- Post	-3.03	1.28	-5.54--0.53
- Follow-up	-2.88	1.44	-5.70--0.07
**Mental Health**			
Behavioral Activation for Depression (BADS)			
- Post	14.78	3.12	8.75-20.92
- Follow-up	14.53	4.40	6.11-23.20
Perceived Stress (PSS-10)			
- Post	-3.08	0.88	-4.81--1.36
- Follow-up	-2.34	1.22	-4.73-0.04
Anxiety (STAI-6)			
- Post	-1.26	0.57	-2.36--0.13
- Follow-up	-0.59	0.69	-1.98-0.73
Worrying (AWQ)			
- Post	-1.65	0.74	-3.09--0.19
- Follow-up	-1.27	0.94	-3.12-0.57
Emotional Exhaustion (MBI-S)			
- Post	-0.42	0.84	-2.07-1.24
- Follow-up	-3.69	0.92	-5.49--1.87
**Academic Outcomes**			
Work Impairment (WIS)			
- Post	-1.66	0.88	-3.38-0.06
- Follow-up	-2.81	1.10	-4.98--0.68
Work Output (WOS)			
- Post	7.30	3.89	-0.34-14.95
- Follow-up	7.94	5.32	-2.83-17.8
Work Cutback (PS-S)			
- Post	-2.13	1.59	-5.26-0.99
- Follow-up	-5.22	2.37	-9.85--0.59
College Self-Efficacy (CSEI)			
- Post	-1.18	1.81	-4.71-2.39
- Follow-up	-0.20	2.52	-5.13-4.74
**Risk and Protective Factors**			
Resilience (CD-RISC)			
- Post	0.45	0.23	0.00-0.89
- Follow-up	0.84	0.29	0.29-1.41

Emotion Regulation Competencies (SEK-27)			
- Post	8.50	1.72	5.14-11.84
- Follow-up	4.10	3.46	-2.48-10.99
Self-Compassion (SCS-D)			
- Post	4.74	1.00	2.76-6.70
- Follow-up	5.45	1.24	3.00-7.87
Self-Esteem (RSES)			
- Post	1.76	0.58	0.62-2.92
- Follow-up	1.22	1.43	-1.76-3.88
Negative Beliefs about Stress (BASS)			
- Post	-0.41	0.51	-1.4-0.58
- Follow-up	-1.75	0.59	-2.91--0.60
Positive Beliefs about Stress (BASS)			
- Post	0.82	0.36	0.11-1.52
- Follow-up	0.75	0.40	-0.05-1.53
Controllability Beliefs about Stress (BASS)			
- Post	1.13	0.25	0.63-1.63
- Follow-up	1.53	0.31	0.91-2.12

Table S3. *Estimated Outcome Intercorrelations (joint Bayesian model)*

**Post-Test (7 weeks)**	Depression	Activation	Stress	Emotional Exhaustion	Positive Beliefs about Stress	Negative Beliefs about Stress	Controllability Beliefs about Stress	Anxiety	Resilience	Worrying	College Self-Efficacy	Self-Esteem	Emotion Regulation	Self-Compassion	Work Impairment	Work Output	Work Cutback
Depression	1.00	-0.48	0.57	0.19	-0.12	0.14	-0.18	0.30	-0.28	0.25	-0.09	-0.44	-0.35	-0.19	0.27	-0.28	0.14
Activation	-0.48	1.00	-0.53	-0.26	0.14	-0.16	0.19	-0.37	0.23	-0.21	0.13	0.41	0.34	0.21	-0.39	0.32	-0.18
Stress	0.57	-0.53	1.00	0.26	-0.21	0.25	-0.35	0.41	-0.22	0.25	-0.14	-0.38	-0.44	-0.25	0.33	-0.27	0.04
Emotional Exhaustion	0.19	-0.26	0.26	1.00	-0.11	0.13	-0.11	0.14	-0.05	0.25	-0.23	-0.04	-0.14	-0.03	0.25	-0.10	0.05
Positive Beliefs about Stress	-0.12	0.14	-0.21	-0.11	1.00	-0.36	0.28	0.04	0.17	0.02	0.09	-0.01	0.13	0.03	0.00	0.10	0.01
Negative Beliefs about Stress	0.14	-0.16	0.25	0.13	-0.36	1.00	-0.08	0.06	-0.10	0.02	-0.14	-0.07	-0.09	-0.10	0.14	-0.22	0.10
Controllability Beliefs about Stress	-0.18	0.19	-0.35	-0.11	0.28	-0.08	1.00	-0.14	0.17	-0.06	0.14	0.21	0.28	0.17	-0.06	0.06	0.05
Anxiety	0.30	-0.37	0.41	0.14	0.04	0.06	-0.14	1.00	-0.12	0.17	-0.11	-0.15	-0.21	-0.23	0.34	-0.14	0.07
Resilience	-0.28	0.23	-0.22	-0.05	0.17	-0.10	0.17	-0.12	1.00	-0.15	0.07	0.30	0.24	0.11	-0.12	0.15	0.03
Worrying	0.25	-0.21	0.25	0.25	0.02	0.02	-0.06	0.17	-0.15	1.00	-0.04	-0.31	-0.23	-0.11	0.15	-0.03	0.04
College Self-Efficacy	-0.09	0.13	-0.14	-0.23	0.09	-0.14	0.14	-0.11	0.07	-0.04	1.00	-0.02	0.16	-0.04	-0.23	0.16	-0.03
Self-Esteem	-0.44	0.41	-0.38	-0.04	-0.01	-0.07	0.21	-0.15	0.30	-0.31	-0.02	1.00	0.31	0.39	-0.26	0.12	-0.03
Emotion Regulation	-0.35	0.34	-0.44	-0.14	0.13	-0.09	0.28	-0.21	0.24	-0.23	0.16	0.31	1.00	0.24	-0.21	0.13	0.00
Self-Compassion	-0.19	0.21	-0.25	-0.03	0.03	-0.10	0.17	-0.23	0.11	-0.11	-0.04	0.39	0.24	1.00	-0.29	0.14	-0.03
Work Impairment	0.27	-0.39	0.33	0.25	0.00	0.14	-0.06	0.34	-0.12	0.15	-0.23	-0.26	-0.21	-0.29	1.00	-0.50	0.26
Work Output	-0.28	0.32	-0.27	-0.10	0.10	-0.22	0.06	-0.14	0.15	-0.03	0.16	0.12	0.13	0.14	-0.50	1.00	-0.35
Work Cutback	0.14	-0.18	0.04	0.05	0.01	0.10	0.05	0.07	0.03	0.04	-0.03	-0.03	0.00	-0.03	0.26	-0.35	1.00
Depression	1.00	-0.30	0.49	0.23	-0.12	0.14	-0.15	0.58	-0.09	0.26	-0.07	-0.05	0.00	-0.21	0.25	-0.17	0.20
Activation	-0.30	1.00	-0.20	0.00	-0.09	-0.01	0.25	-0.18	0.37	-0.05	0.31	0.30	0.38	0.38	-0.52	0.34	-0.17
Stress	0.49	-0.20	1.00	0.32	-0.11	0.13	-0.18	0.54	0.05	0.39	0.06	0.10	0.16	-0.10	0.21	-0.23	0.01
Emotional Exhaustion	0.23	0.00	0.32	1.00	-0.01	0.08	0.01	0.18	0.21	0.28	0.09	0.19	0.17	0.05	0.13	-0.19	-0.05
Positive Beliefs about Stress	-0.12	-0.09	-0.11	-0.01	1.00	-0.34	0.20	-0.10	0.18	0.00	-0.16	-0.12	-0.12	0.04	0.01	0.01	0.02
Negative Beliefs about Stress	0.14	-0.01	0.13	0.08	-0.34	1.00	0.00	0.14	-0.02	0.00	0.06	0.17	0.18	-0.04	-0.01	0.02	0.05
Controllability Beliefs about Stress	-0.15	0.25	-0.18	0.01	0.20	0.00	1.00	-0.01	0.21	-0.02	0.04	0.08	0.09	0.24	-0.12	0.11	-0.01
Anxiety	0.58	-0.18	0.54	0.18	-0.10	0.14	-0.01	1.00	-0.02	0.26	0.03	0.06	0.08	-0.09	0.22	-0.16	0.05

**Follow-up (3 months)**	Depression	Activation	Stress	Emotional Exhaustion	Positive Beliefs about Stress	Negative Beliefs about Stress	Controllability Beliefs about Stress	Anxiety	Resilience	Worrying	College Self-Efficacy	Self-Esteem	Emotion Regulation	Self-Compassion	Work Impairment	Work Output	Work Cutback
Resilience	-0.09	0.37	0.05	0.21	0.18	-0.02	0.21	-0.02	1.00	0.24	0.32	0.29	0.36	0.36	-0.27	0.13	-0.15
Worrying	0.26	-0.05	0.39	0.28	0.00	0.00	-0.02	0.26	0.24	1.00	0.15	0.06	0.08	0.02	0.07	-0.09	-0.12
College Self-Efficacy	-0.07	0.31	0.06	0.09	-0.16	0.06	0.04	0.03	0.32	0.15	1.00	0.44	0.50	0.23	-0.18	0.23	-0.11
Self-Esteem	-0.05	0.30	0.10	0.19	-0.12	0.17	0.08	0.06	0.29	0.06	0.44	1.00	0.52	0.29	-0.26	0.14	0.01
Emotion Regulation	0.00	0.38	0.16	0.17	-0.12	0.18	0.09	0.08	0.36	0.08	0.50	0.52	1.00	0.34	-0.12	0.06	-0.08
Self-Compassion	-0.21	0.38	-0.10	0.05	0.04	-0.04	0.24	-0.09	0.36	0.02	0.23	0.29	0.34	1.00	-0.29	0.09	-0.19
Work Impairment	0.25	-0.52	0.21	0.13	0.01	-0.01	-0.12	0.22	-0.27	0.07	-0.18	-0.26	-0.12	-0.29	1.00	-0.44	0.12
Work Output	-0.17	0.34	-0.23	-0.19	0.01	0.02	0.11	-0.16	0.13	-0.09	0.23	0.14	0.06	0.09	-0.44	1.00	-0.16
Work Cutback	0.20	-0.17	0.01	-0.05	0.02	0.05	-0.01	0.05	-0.15	-0.12	-0.11	0.01	-0.08	-0.19	0.12	-0.16	1.00

Table S4. *Between-group Effect Sizes and Results of Analyses of Covariance (ANCOVA) in Participants with values of CES-D ≥ 20 for the Primary and Secondary Outcomes at Post-Test and Follow-Up.*

Outcome and Assessment Point	Effect Size		ANCOVA	
	d	95%CI	*F*	*p*
**Primary Outcome**				
Depression (CES-D)				
- Post	0.37	0.02-0.72	4.52	0.035
- Follow-up	0.34	-0.03-0.70	3.22	0.074
**Mental Health**				
Behavioral Activation for Depression (BADS)				
- Post	0.68	0.33-1.04	15.26	<0.001
- Follow-up	0.48	0.10-0.86	5.79	0.026
Perceived Stress (PSS-10)				
- Post	0.52	0.18-0.87	9.57	0.003
- Follow-up	0.40	0.04-0.76	5.57	0.019
Anxiety (STAI-6)				
- Post	0.33	-0.09-0.74	1.93	0.188
- Follow-up	0.14	-0.28-0.56	0.04	1.000
Worrying (AWQ)				
- Post	0.33	-0.04-0.70	3.49	0.065
- Follow-up	0.39	-0.01-0.78	3.72	0.063
Emotional Exhaustion (MBI-S)				
- Post	0.02	-0.34-0.38	0.02	1.000
- Follow-up	0.65	0.32-0.98	20.75	<0.001
**Academic Outcomes**				
Work Impairment (WIS)				
- Post	0.22	-0.11-0.55	2.60	0.107
- Follow-up	0.36	-0.02-0.75	3.81	0.061
Work Output (WOS)				
- Post	0.38	0.07-0.70	5.86	0.016
- Follow-up	0.32	-0.17-0.81	0.86	0.390
Work Cutback (PS-S)				
- Post	0.26	-0.10-0.62	1.91	0.171
- Follow-up	0.33	-0.02-0.67	3.43	0.065
College Self-Efficacy (CSEI)				
- Post	0.02	-0.34-0.37	-0.01	1.000
- Follow-up	0.00	-0.34-0.34	0.00	0.949
**Risk and Protective Factors**				

Resilience (CD-RISC)				
- Post	0.23	-0.14-0.61	1.42	0.241
- Follow-up	0.27	-0.06-0.61	3.13	0.077
Emotion Regulation Competencies (SEK-27)				
- Post	0.65	0.32-0.98	19.36	<0.001
- Follow-up	0.14	-0.20-0.48	0.52	0.471
Self-Compassion (SCS-D)				
- Post	0.60	0.26-0.94	17.32	<0.001
- Follow-up	0.61	0.21-1.00	10.01	0.014
Self-Esteem (RSES)				
- Post	0.35	0.00-0.70	8.53	0.004
- Follow-up	0.12	-0.21-0.45	0.38	0.536
Negative Beliefs about Stress (BASS)				
- Post	0.06	-0.28-0.40	0.12	0.727
- Follow-up	0.37	-0.03-0.77	3.29	0.086
Positive Beliefs about Stress (BASS)				
- Post	0.21	-0.13-0.54	2.14	0.143
- Follow-up	0.23	-0.12-0.57	2.25	0.134
Controllability Beliefs about Stress (BASS)				
- Post	0.73	0.39-1.07	20.15	<0.001
- Follow-up	0.88	0.52-1.25	16.73	0.003

Note: These analyses are based on the n=138 (69%) participants with values of CES-D $\geq$ 20 at baseline (IG: n=66/66%; CG: n=72/72%).

Table S5. *Intervention Usefulness, Difficulty and Duration Ratings in the Intervention Group.*

Module and Criterion	Mean Rating	*n*
**Module 1**		
- Usefulness (1-5)	3.95	88
- Difficulty (1-5)	2.13	88
- Duration (1-4)	2.32	87
**Module 2**		
- Usefulness (1-5)	4.18	82
- Difficulty (1-5)	2.41	83
- Duration (1-4)	2.71	83
**Module 3**		
- Usefulness (1-5)	4.22	74
- Difficulty (1-5)	2.34	73
- Duration (1-4)	3.00	74
**Module 4**		
- Usefulness (1-5)	4.28	72
- Difficulty (1-5)	2.08	72
- Duration (1-4)	2.54	72
**Module 5**		
- Usefulness (1-5)	4.34	65
- Difficulty (1-5)	2.56	66
- Duration (1-4)	2.94	64
**Module 6**		
- Usefulness (1-5)	4.29	63
- Difficulty (1-5)	2.68	63
- Duration (1-4)	3.06	63
**Module 7**		
- Usefulness (1-5)	4.27	60
- Difficulty (1-5)	1.65	60
- Duration (1-4)	2.08	61

Note: Rating options for duration were: *1*=less than ½ hour; *2*= ½ to 1 hour; *3*=1 to 1½ hours; *4*=more than 1½ hours.

Table S6. *Participants' satisfaction with the interventions (assessed at post-test).*

Ratings	Intervention Group (*StudiCare Fernstudierende*)		Active Control Group (Psychoeducation)	
	n	%	*n*	%
Quality of the training rated as excellent or good	78	95.1	69	74.2
Indication that the training was the kind of intervention they wanted to receive (generally or definitely)	78	95.1	32	34.4
Indication that the own needs were almost all or mostly met	73	89	30	32.3
Inclination to recommend the training to a friend in need of similar help	77	93.9	45	48.4
Satisfaction with the amount of help received (mostly or very satisfied)	77	93.9	38	40.9
Indication that the training has helped (a great deal) to deal more effectively with problems	79	96.3	35	37.6
Satisfaction with the training in a general, overall sense (mostly or very satisfied)	78	95.1	32	34.4
Inclination to use the training again if in need for help	70	85.4	43	46.2

Figure S1. *Effect sizes (standardized posterior probability) estimated by the joint Bayesian model at post-test and follow-up*

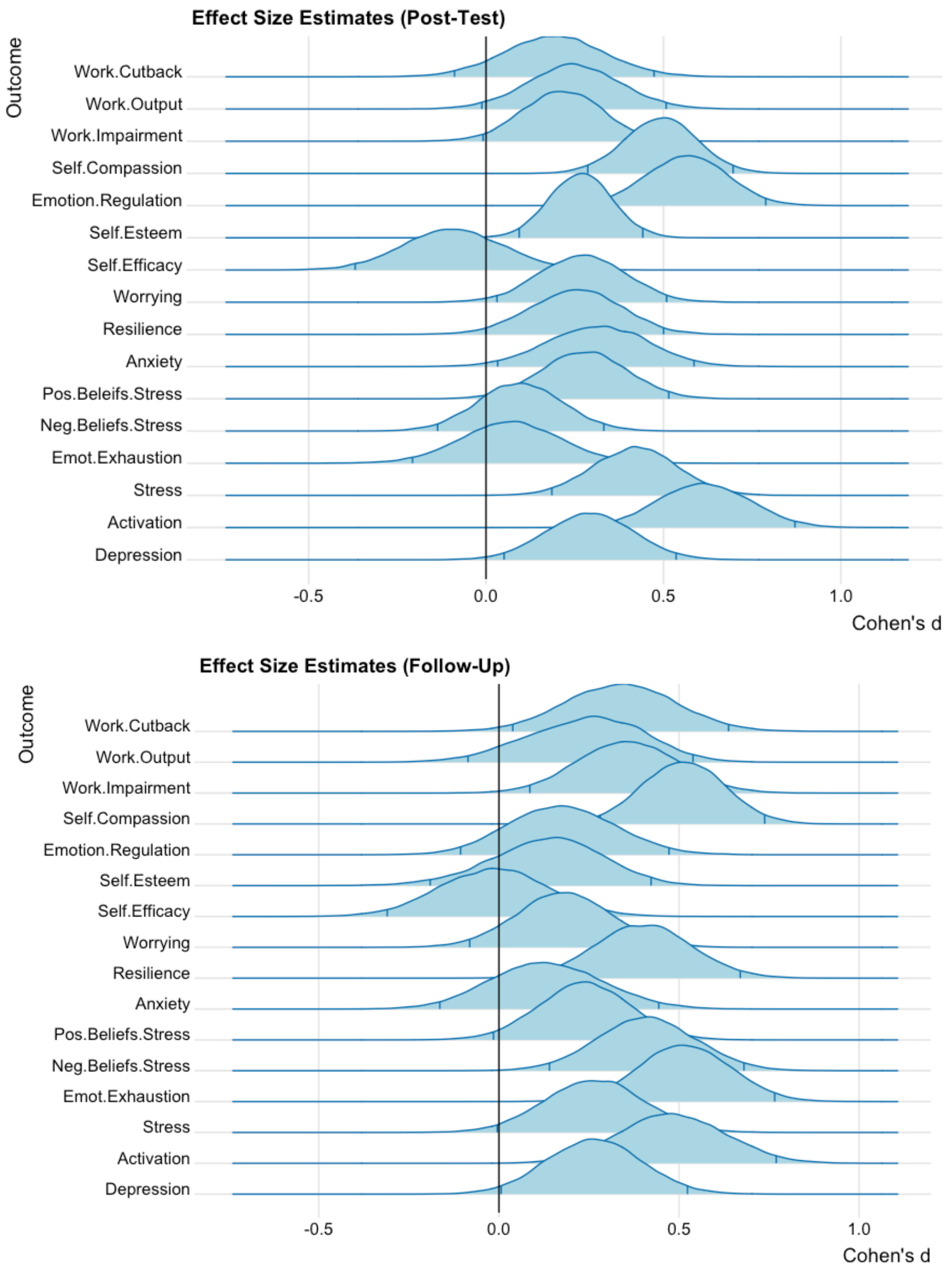

Supplement: Supplementary file 1 — Supplementary material [file mmc1.pdf]
